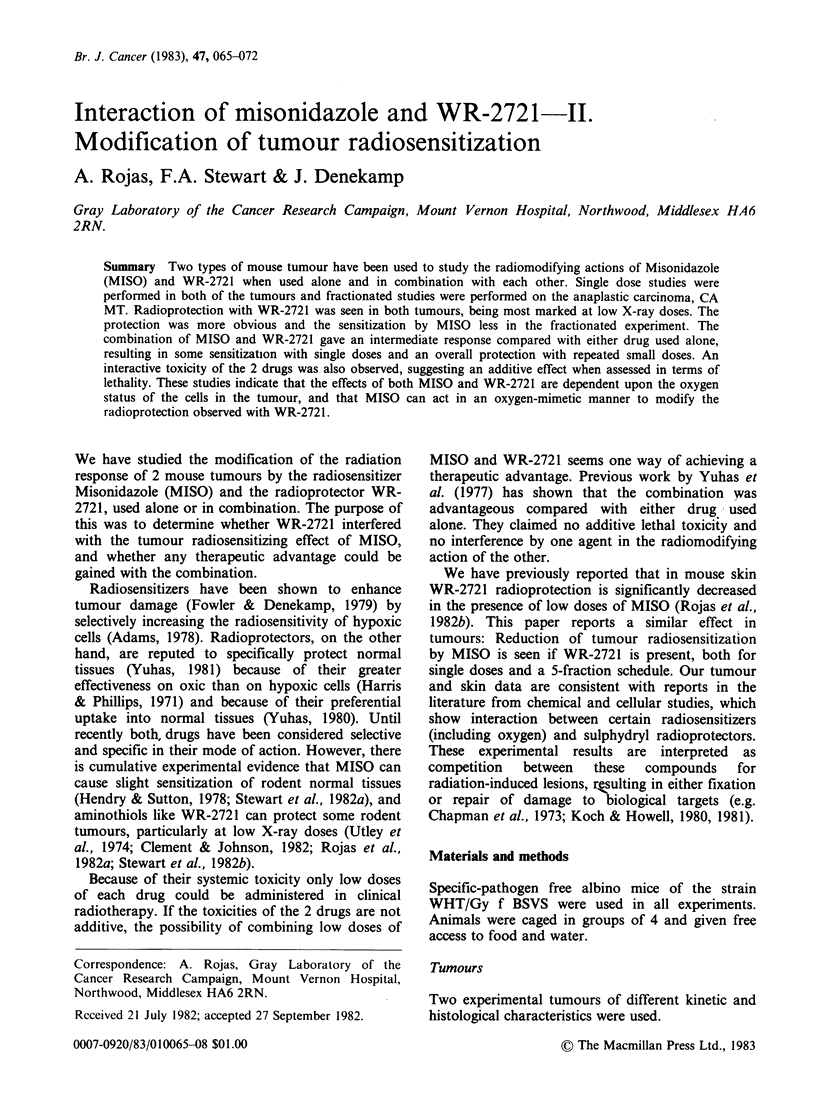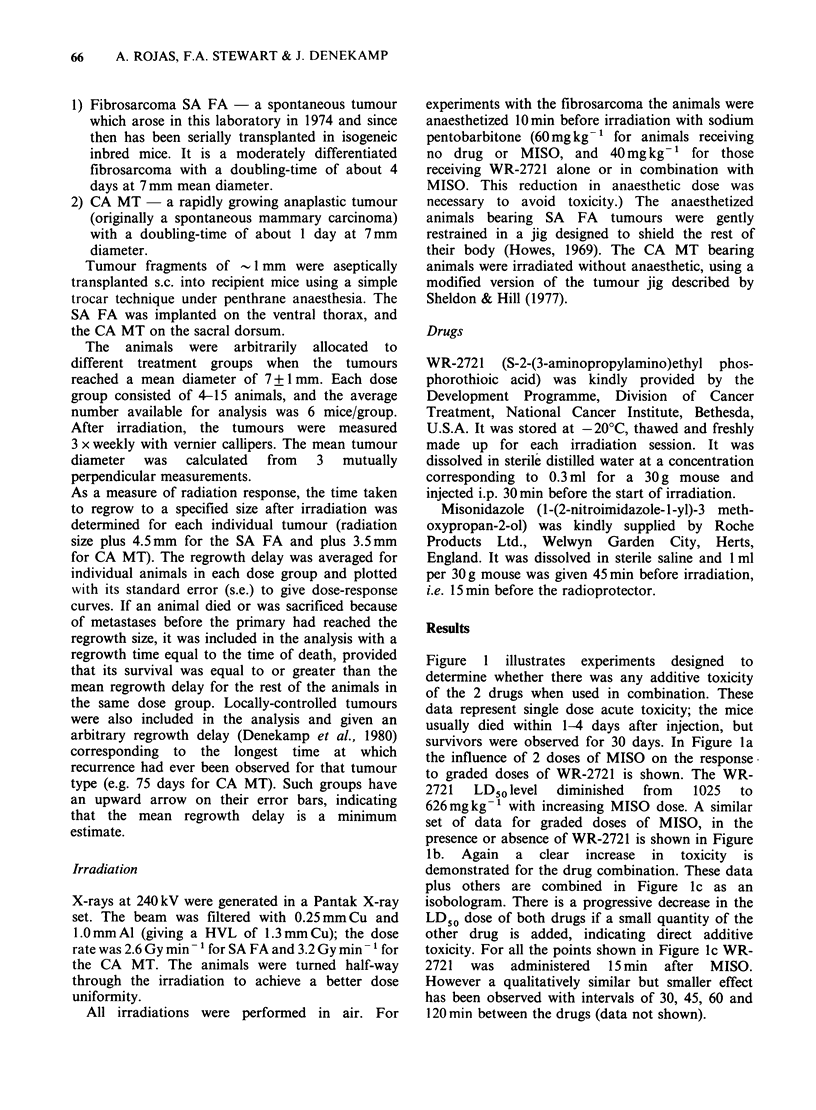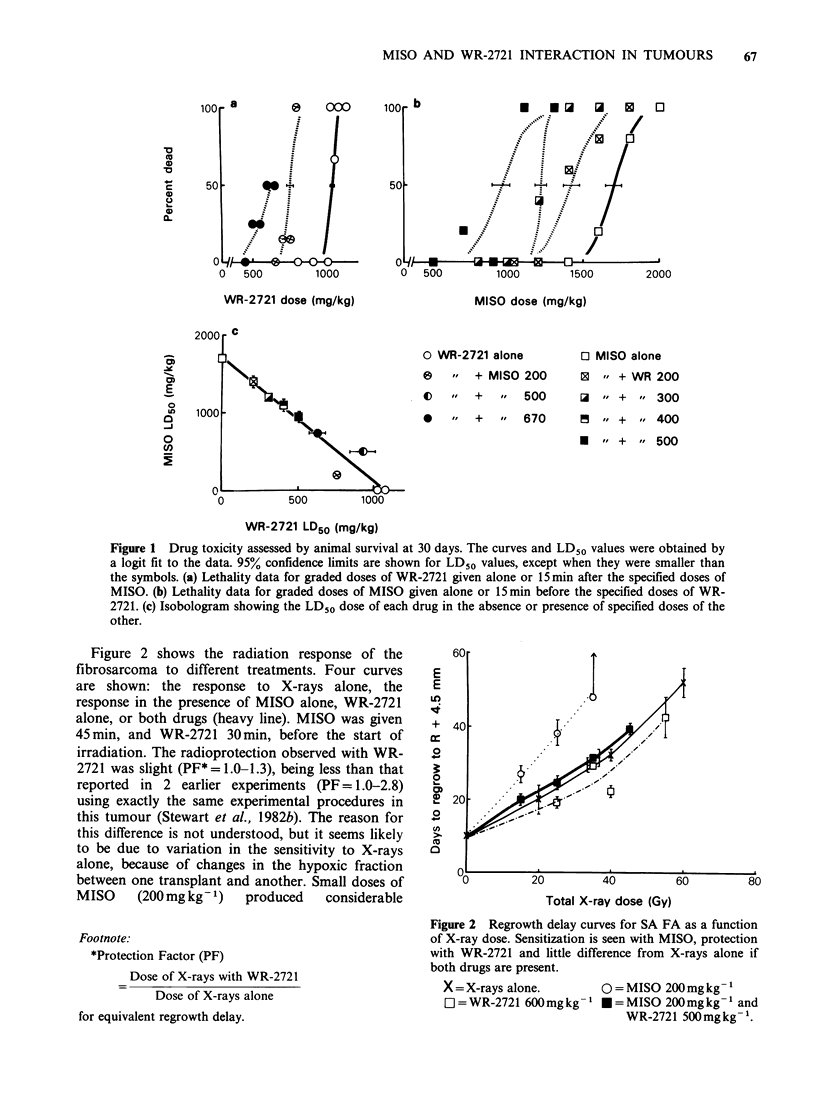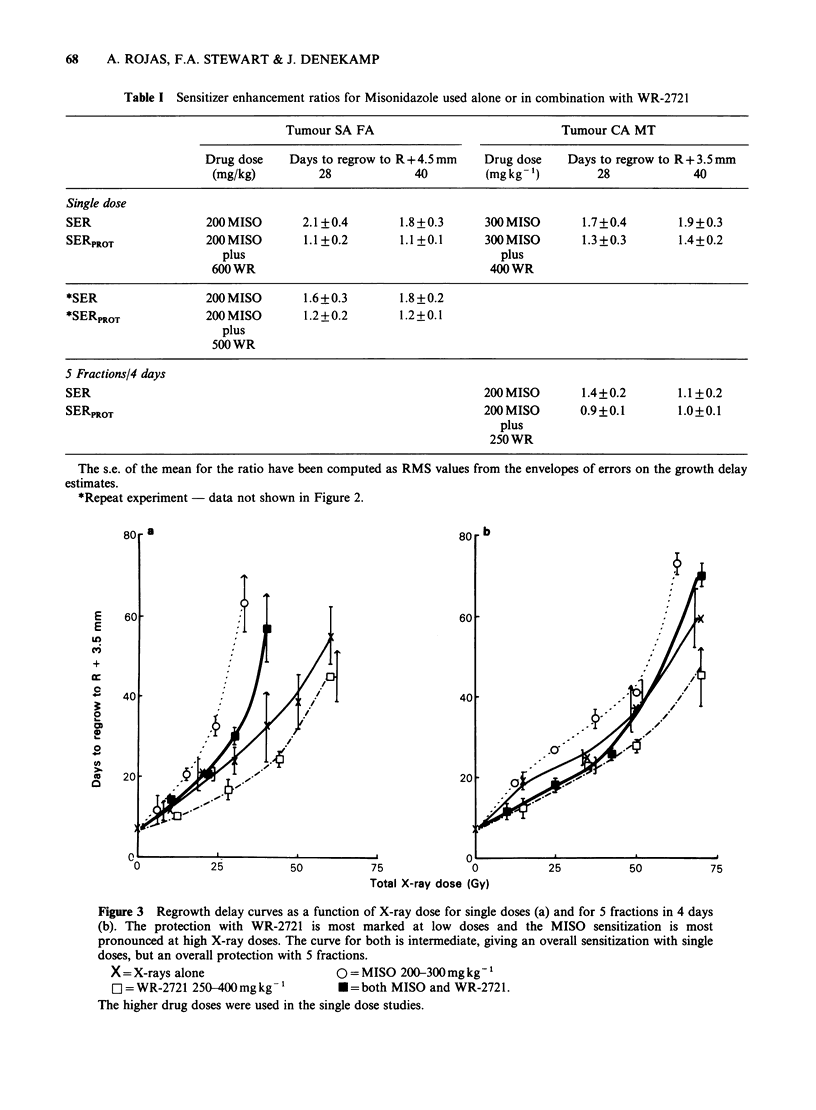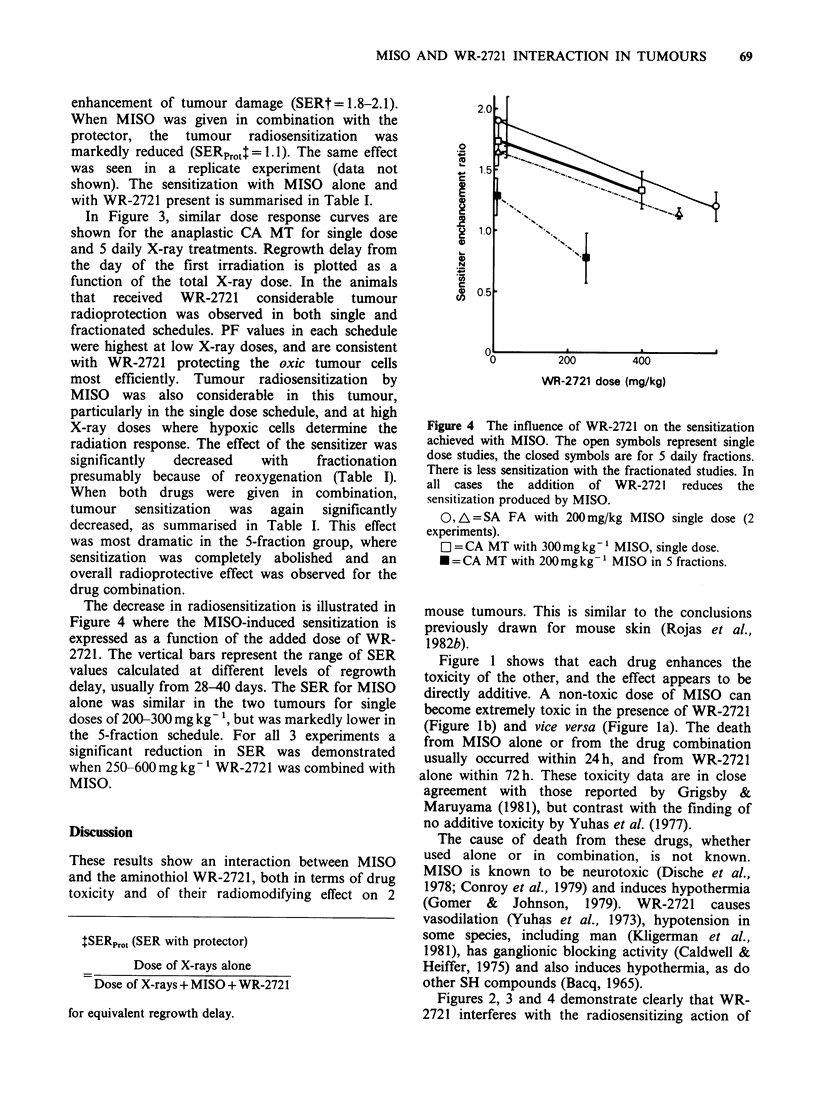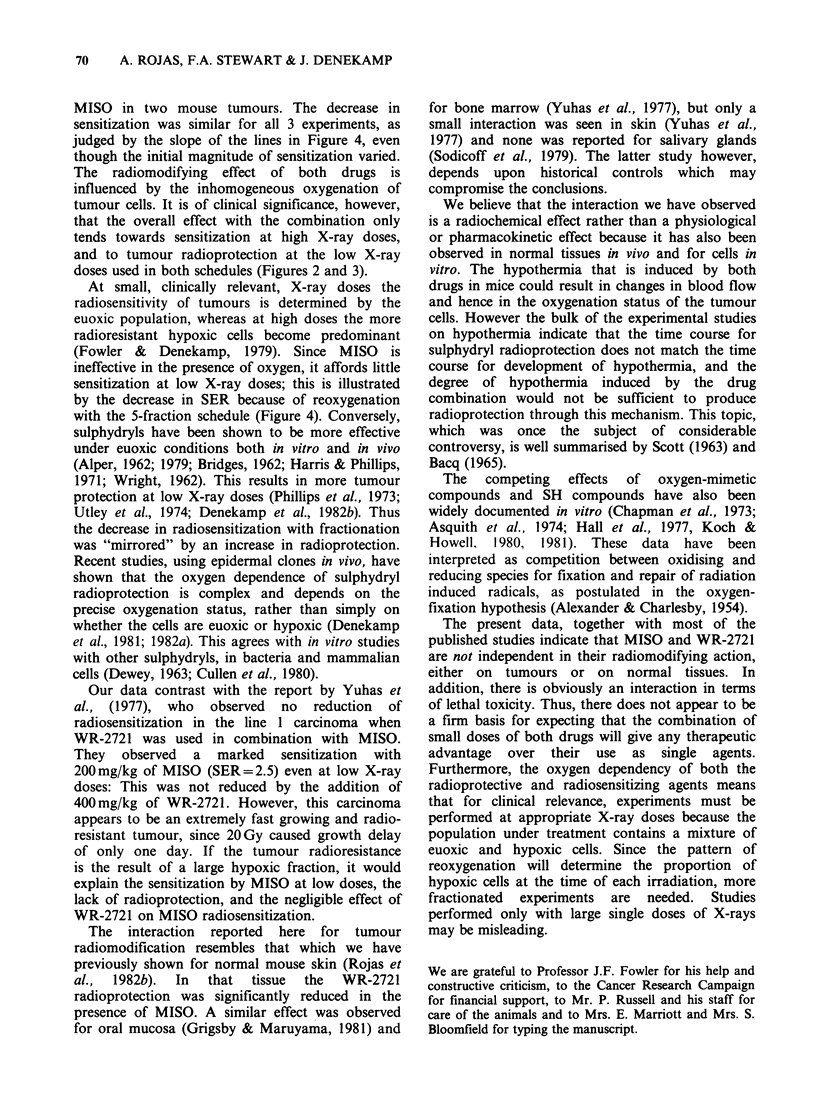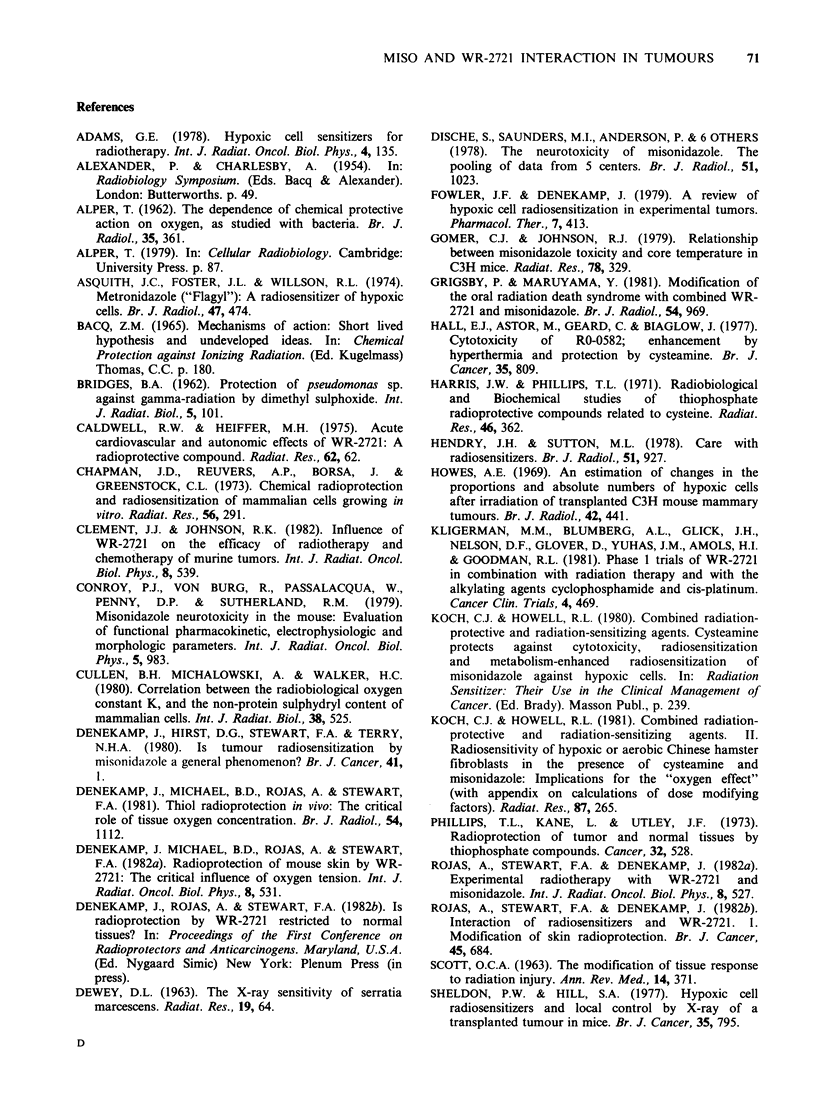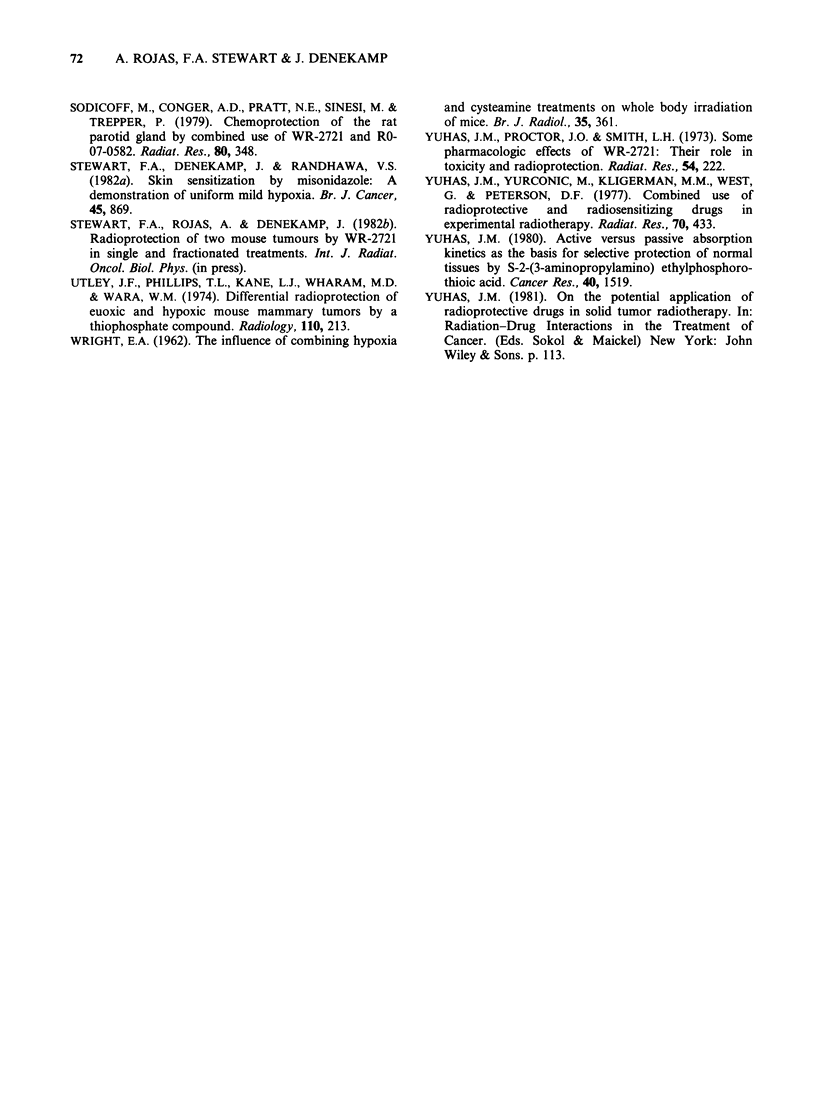# Interaction of misonidazole and WR-2721--II. Modification of tumour radiosensitization.

**DOI:** 10.1038/bjc.1983.8

**Published:** 1983-01

**Authors:** A. Rojas, F. A. Stewart, J. Denekamp

## Abstract

Two types of mouse tumour have been used to study the radiomodifying actions of Misonidazole (MISO) and WR-2721 when used alone and in combination with each other. Single dose studies were performed in both of the tumours and fractionated studies were performed on the anaplastic carcinoma, CA MT. Radioprotection with WR-2721 was seen in both tumours, being most marked at low X-ray doses. The protection was more obvious and the sensitization by MISO less in the fractionated experiment. The combination of MISO and WR-2721 gave an intermediate response compared with either drug used alone, resulting in some sensitization with single doses and an overall protection with repeated small doses. An interactive toxicity of the 2 drugs was also observed, suggesting an additive effect when assessed in terms of lethality. These studies indicate that the effects of both MISO and WR-2721 are dependent upon the oxygen status of the cells in the tumour, and that MISO can act in an oxygen-mimetic manner to modify the radioprotection observed with WR-2721.


Br. J. Cancer (1983), 47, 065–072

### Interaction of misonidazole and WR-2721—II. Modification of tumour radiosensitization

A. Rojas, F.A. Stewart & J. Denekamp

*Gray Laboratory of the Cancer Research Campaign, Mount Vernon Hospital, Northwood, Middlesex HA6 2RN.*

**Summary** Two types of mouse tumour have been used to study the radiomodifying actions of Misonidazole (MISO) and WR-2721 when used alone and in combination with each other. Single dose studies were performed in both of the tumours and fractionated studies were performed on the anaplastic carcinoma, CA MT. Radioprotection with WR-2721 was seen in both tumours, being most marked at low X-ray doses. The protection was more obvious and the sensitization by MISO less in the fractionated experiment. The combination of MISO and WR-2721 gave an intermediate response compared with either drug used alone, resulting in some sensitization with single doses and an overall protection with repeated small doses. An interactive toxicity of the 2 drugs was also observed, suggesting an additive effect when assessed in terms of lethality. These studies indicate that the effects of both MISO and WR-2721 are dependent upon the oxygen status of the cells in the tumour, and that MISO can act in an oxygen-mimetic manner to modify the radioprotection observed with WR-2721.

We have studied the modification of the radiation response of 2 mouse tumours by the radiosensitizer Misonidazole (MISO) and the radioprotector WR-2721, used alone or in combination. The purpose of this was to determine whether WR-2721 interfered with the tumour radiosensitizing effect of MISO, and whether any therapeutic advantage could be gained with the combination.

Radiosensitizers have been shown to enhance tumour damage (Fowler & Denekamp, 1979) by selectively increasing the radiosensitivity of hypoxic cells (Adams, 1978). Radioprotectors, on the other hand, are reputed to specifically protect normal tissues (Yuhas, 1981) because of their greater effectiveness on oxic than on hypoxic cells (Harris & Phillips, 1971) and because of their preferential uptake into normal tissues (Yuhas, 1980). Until recently both drugs have been considered selective and specific in their mode of action. However, there is cumulative experimental evidence that MISO can cause slight sensitization of rodent normal tissues (Hendry & Sutton, 1978; Stewart *et al.*, 1982*a*), and aminothiols like WR-2721 can protect some rodent tumours, particularly at low X-ray doses (Utley *et al.*, 1974; Clement & Johnson, 1982; Rojas *et al.*, 1982*a*; Stewart *et al.*, 1982*b*).

Because of their systemic toxicity only low doses of each drug could be administered in clinical radiotherapy. If the toxicities of the 2 drugs are not additive, the possibility of combining low doses of MISO and WR-2721 seems one way of achieving a therapeutic advantage. Previous work by Yuhas *et al.* (1977) has shown that the combination was advantageous compared with either drug used alone. They claimed no additive lethal toxicity and no interference by one agent in the radiomodifying action of the other.

We have previously reported that in mouse skin WR-2721 radioprotection is significantly decreased in the presence of low doses of MISO (Rojas *et al.*, 1982*b*). This paper reports a similar effect in tumours: Reduction of tumour radiosensitization by MISO is seen if WR-2721 is present, both for single doses and a 5-fraction schedule. Our tumour and skin data are consistent with reports in the literature from chemical and cellular studies, which show interaction between certain radiosensitizers (including oxygen) and sulphydryl radioprotectors. These experimental results are interpreted as competition between these compounds for radiation-induced lesions, resulting in either fixation or repair of damage to biological targets (e.g. Chapman *et al.*, 1973; Koch & Howell, 1980, 1981).

### Materials and methods

Specific-pathogen free albino mice of the strain WHT/Gy f BSVS were used in all experiments. Animals were caged in groups of 4 and given free access to food and water.

### *Tumours*

Two experimental tumours of different kinetic and histological characteristics were used.

Correspondence: A. Rojas, Gray Laboratory of the Cancer Research Campaign, Mount Vernon Hospital, Northwood, Middlesex HA6 2RN.

Received 21 July 1982; accepted 27 September 1982.

0007-0920/83/010065–08 $01.00

© The Macmillan Press Ltd., 1983

1) Fibrosarcoma SA FA — a spontaneous tumour which arose in this laboratory in 1974 and since then has been serially transplanted in isogeneic inbred mice. It is a moderately differentiated fibrosarcoma with a doubling-time of about 4 days at 7 mm mean diameter.
2) CA MT — a rapidly growing anaplastic tumour (originally a spontaneous mammary carcinoma) with a doubling-time of about 1 day at 7 mm diameter.

Tumour fragments of ~1 mm were aseptically transplanted s.c. into recipient mice using a simple trocar technique under penthrane anaesthesia. The SA FA was implanted on the ventral thorax, and the CA MT on the sacral dorsum.

The animals were arbitrarily allocated to different treatment groups when the tumours reached a mean diameter of $7 \pm 1$ mm. Each dose group consisted of 4–15 animals, and the average number available for analysis was 6 mice/group. After irradiation, the tumours were measured 3 x weekly with vernier callipers. The mean tumour diameter was calculated from 3 mutually perpendicular measurements.

As a measure of radiation response, the time taken to regrow to a specified size after irradiation was determined for each individual tumour (radiation size plus 4.5 mm for the SA FA and plus 3.5 mm for CA MT). The regrowth delay was averaged for individual animals in each dose group and plotted with its standard error (s.e.) to give dose-response curves. If an animal died or was sacrificed because of metastases before the primary had reached the regrowth size, it was included in the analysis with a regrowth time equal to the time of death, provided that its survival was equal to or greater than the mean regrowth delay for the rest of the animals in the same dose group. Locally-controlled tumours were also included in the analysis and given an arbitrary regrowth delay (Denekamp *et al.*, 1980) corresponding to the longest time at which recurrence had ever been observed for that tumour type (e.g. 75 days for CA MT). Such groups have an upward arrow on their error bars, indicating that the mean regrowth delay is a minimum estimate.

### *Irradiation*

X-rays at 240 kV were generated in a Pantak X-ray set. The beam was filtered with 0.25 mm Cu and 1.0 mm Al (giving a HVL of 1.3 mm Cu); the dose rate was 2.6 Gy $min^{-1}$ for SA FA and 3.2 Gy $min^{-1}$ for the CA MT. The animals were turned half-way through the irradiation to achieve a better dose uniformity.

All irradiations were performed in air. For experiments with the fibrosarcoma the animals were anaesthetized 10 min before irradiation with sodium pentobarbitone (60 mg $kg^{-1}$ for animals receiving no drug or MISO, and 40 mg $kg^{-1}$ for those receiving WR-2721 alone or in combination with MISO. This reduction in anaesthetic dose was necessary to avoid toxicity.) The anaesthetized animals bearing SA FA tumours were gently restrained in a jig designed to shield the rest of their body (Howes, 1969). The CA MT bearing animals were irradiated without anaesthetic, using a modified version of the tumour jig described by Sheldon & Hill (1977).

### *Drugs*

WR-2721 (S-2-(3-aminopropylamino)ethyl phosphorothioic acid) was kindly provided by the Development Programme, Division of Cancer Treatment, National Cancer Institute, Bethesda, U.S.A. It was stored at −20°C, thawed and freshly made up for each irradiation session. It was dissolved in sterile distilled water at a concentration corresponding to 0.3 ml for a 30 g mouse and injected i.p. 30 min before the start of irradiation.

Misonidazole (1-(2-nitroimidazole-1-yl)-3 methoxypropan-2-ol) was kindly supplied by Roche Products Ltd., Welwyn Garden City, Herts, England. It was dissolved in sterile saline and 1 ml per 30 g mouse was given 45 min before irradiation, *i.e.* 15 min before the radioprotector.

### Results

Figure 1 illustrates experiments designed to determine whether there was any additive toxicity of the 2 drugs when used in combination. These data represent single dose acute toxicity; the mice usually died within 1–4 days after injection, but survivors were observed for 30 days. In Figure 1a the influence of 2 doses of MISO on the response to graded doses of WR-2721 is shown. The WR-2721 $LD_{50}$ level diminished from 1025 to 626 mg $kg^{-1}$ with increasing MISO dose. A similar set of data for graded doses of MISO, in the presence or absence of WR-2721 is shown in Figure 1b. Again a clear increase in toxicity is demonstrated for the drug combination. These data plus others are combined in Figure 1c as an isobologram. There is a progressive decrease in the $LD_{50}$ dose of both drugs if a small quantity of the other drug is added, indicating direct additive toxicity. For all the points shown in Figure 1c WR-2721 was administered 15 min after MISO. However a qualitatively similar but smaller effect has been observed with intervals of 30, 45, 60 and 120 min between the drugs (data not shown).

**Figure 1** Drug toxicity assessed by animal survival at 30 days. The curves and $LD_{50}$ values were obtained by a logit fit to the data. 95% confidence limits are shown for $LD_{50}$ values, except when they were smaller than the symbols. (**a**) Lethality data for graded doses of WR-2721 given alone or 15 min after the specified doses of MISO. (**b**) Lethality data for graded doses of MISO given alone or 15 min before the specified doses of WR-2721. (**c**) Isobologram showing the $LD_{50}$ dose of each drug in the absence or presence of specified doses of the other.

Figure 2 shows the radiation response of the fibrosarcoma to different treatments. Four curves are shown: the response to X-rays alone, the response in the presence of MISO alone, WR-2721 alone, or both drugs (heavy line). MISO was given 45 min, and WR-2721 30 min, before the start of irradiation. The radioprotection observed with WR-2721 was slight (PF* = 1.0–1.3), being less than that reported in 2 earlier experiments (PF = 1.0–2.8) using exactly the same experimental procedures in this tumour (Stewart *et al.*, 1982*b*). The reason for this difference is not understood, but it seems likely to be due to variation in the sensitivity to X-rays alone, because of changes in the hypoxic fraction between one transplant and another. Small doses of MISO (200 mg $kg^{-1}$) produced considerable

**Figure 2** Regrowth delay curves for SA FA as a function of X-ray dose. Sensitization is seen with MISO, protection with WR-2721 and little difference from X-rays alone if both drugs are present.

X = X-rays alone. ○ = MISO 200 mg $kg^{-1}$

□ = WR-2721 600 mg $kg^{-1}$ ■ = MISO 200 mg $kg^{-1}$ and WR-2721 500 mg $kg^{-1}$.

*Footnote:*

*Protection Factor (PF)

$$= \frac{\text{Dose of X-rays with WR-2721}}{\text{Dose of X-rays alone}}$$

for equivalent regrowth delay.

**Table I** Sensitizer enhancement ratios for Misonidazole used alone or in combination with WR-2721

| | Tumour SA FA | | | Tumour CA MT | | |
|---|---|---|---|---|---|---|
| | Drug dose (mg/kg) | Days to regrow to R+4.5 mm 28 | 40 | Drug dose (mg kg$^{-1}$) | Days to regrow to R+3.5 mm 28 | 40 |
| *Single dose* | | | | | | |
| SER | 200 MISO | 2.1±0.4 | 1.8±0.3 | 300 MISO | 1.7±0.4 | 1.9±0.3 |
| $SER_{PROT}$ | 200 MISO plus 600 WR | 1.1±0.2 | 1.1±0.1 | 300 MISO plus 400 WR | 1.3±0.3 | 1.4±0.2 |
| *SER | 200 MISO | 1.6±0.3 | 1.8±0.2 | | | |
| *$SER_{PROT}$ | 200 MISO plus 500 WR | 1.2±0.2 | 1.2±0.1 | | | |
| *5 Fractions/4 days* | | | | | | |
| SER | | | | 200 MISO | 1.4±0.2 | 1.1±0.2 |
| $SER_{PROT}$ | | | | 200 MISO plus 250 WR | 0.9±0.1 | 1.0±0.1 |

The s.e. of the mean for the ratio have been computed as RMS values from the envelopes of errors on the growth delay estimates.

*Repeat experiment — data not shown in Figure 2.

**Figure 3** Regrowth delay curves as a function of X-ray dose for single doses (a) and for 5 fractions in 4 days (b). The protection with WR-2721 is most marked at low doses and the MISO sensitization is most pronounced at high X-ray doses. The curve for both is intermediate, giving an overall sensitization with single doses, but an overall protection with 5 fractions.

X = X-rays alone ○ = MISO 200–300 mg kg$^{-1}$

□ = WR-2721 250–400 mg kg$^{-1}$ ■ = both MISO and WR-2721.

The higher drug doses were used in the single dose studies.

enhancement of tumour damage (SER† = 1.8–2.1). When MISO was given in combination with the protector, the tumour radiosensitization was markedly reduced ($SER_{Prot}$‡ = 1.1). The same effect was seen in a replicate experiment (data not shown). The sensitization with MISO alone and with WR-2721 present is summarised in Table I.

In Figure 3, similar dose response curves are shown for the anaplastic CA MT for single dose and 5 daily X-ray treatments. Regrowth delay from the day of the first irradiation is plotted as a function of the total X-ray dose. In the animals that received WR-2721 considerable tumour radioprotection was observed in both single and fractionated schedules. PF values in each schedule were highest at low X-ray doses, and are consistent with WR-2721 protecting the *oxic* tumour cells most efficiently. Tumour radiosensitization by MISO was also considerable in this tumour, particularly in the single dose schedule, and at high X-ray doses where hypoxic cells determine the radiation response. The effect of the sensitizer was significantly decreased with fractionation presumably because of reoxygenation (Table I). When both drugs were given in combination, tumour sensitization was again significantly decreased, as summarised in Table I. This effect was most dramatic in the 5-fraction group, where sensitization was completely abolished and an overall radioprotective effect was observed for the drug combination.

The decrease in radiosensitization is illustrated in Figure 4 where the MISO-induced sensitization is expressed as a function of the added dose of WR-2721. The vertical bars represent the range of SER values calculated at different levels of regrowth delay, usually from 28–40 days. The SER for MISO alone was similar in the two tumours for single doses of 200–300 mg kg$^{-1}$, but was markedly lower in the 5-fraction schedule. For all 3 experiments a significant reduction in SER was demonstrated when 250–600 mg kg$^{-1}$ WR-2721 was combined with MISO.

### Discussion

These results show an interaction between MISO and the aminothiol WR-2721, both in terms of drug toxicity and of their radiomodifying effect on 2

‡$SER_{Prot}$ (SER with protector)

$$= \frac{\text{Dose of X-rays alone}}{\text{Dose of X-rays} + \text{MISO} + \text{WR-2721}}$$

for equivalent regrowth delay.

**Figure 4** The influence of WR-2721 on the sensitization achieved with MISO. The open symbols represent single dose studies, the closed symbols are for 5 daily fractions. There is less sensitization with the fractionated studies. In all cases the addition of WR-2721 reduces the sensitization produced by MISO.

○, △ = SA FA with 200 mg/kg MISO single dose (2 experiments).

□ = CA MT with 300 mg kg$^{-1}$ MISO, single dose.

■ = CA MT with 200 mg kg$^{-1}$ MISO in 5 fractions.

mouse tumours. This is similar to the conclusions previously drawn for mouse skin (Rojas *et al.*, 1982*b*).

Figure 1 shows that each drug enhances the toxicity of the other, and the effect appears to be directly additive. A non-toxic dose of MISO can become extremely toxic in the presence of WR-2721 (Figure 1b) and *vice versa* (Figure 1a). The death from MISO alone or from the drug combination usually occurred within 24 h, and from WR-2721 alone within 72 h. These toxicity data are in close agreement with those reported by Grigsby & Maruyama (1981), but contrast with the finding of no additive toxicity by Yuhas *et al.* (1977).

The cause of death from these drugs, whether used alone or in combination, is not known. MISO is known to be neurotoxic (Dische *et al.*, 1978; Conroy *et al.*, 1979) and induces hypothermia (Gomer & Johnson, 1979). WR-2721 causes vasodilation (Yuhas *et al.*, 1973), hypotension in some species, including man (Kligerman *et al.*, 1981), has ganglionic blocking activity (Caldwell & Heiffer, 1975) and also induces hypothermia, as do other SH compounds (Bacq, 1965).

Figures 2, 3 and 4 demonstrate clearly that WR-2721 interferes with the radiosensitizing action of

MISO in two mouse tumours. The decrease in sensitization was similar for all 3 experiments, as judged by the slope of the lines in Figure 4, even though the initial magnitude of sensitization varied. The radiomodifying effect of both drugs is influenced by the inhomogeneous oxygenation of tumour cells. It is of clinical significance, however, that the overall effect with the combination only tends towards sensitization at high X-ray doses, and to tumour radioprotection at the low X-ray doses used in both schedules (Figures 2 and 3).

At small, clinically relevant, X-ray doses the radiosensitivity of tumours is determined by the euoxic population, whereas at high doses the more radioresistant hypoxic cells become predominant (Fowler & Denekamp, 1979). Since MISO is ineffective in the presence of oxygen, it affords little sensitization at low X-ray doses; this is illustrated by the decrease in SER because of reoxygenation with the 5-fraction schedule (Figure 4). Conversely, sulphydryls have been shown to be more effective under euoxic conditions both *in vitro* and *in vivo* (Alper, 1962; 1979; Bridges, 1962; Harris & Phillips, 1971; Wright, 1962). This results in more tumour protection at low X-ray doses (Phillips *et al.*, 1973; Utley *et al.*, 1974; Denekamp *et al.*, 1982*b*). Thus the decrease in radiosensitization with fractionation was "mirrored" by an increase in radioprotection. Recent studies, using epidermal clones *in vivo*, have shown that the oxygen dependence of sulphydryl radioprotection is complex and depends on the precise oxygenation status, rather than simply on whether the cells are euoxic or hypoxic (Denekamp *et al.*, 1981; 1982*a*). This agrees with *in vitro* studies with other sulphydryls, in bacteria and mammalian cells (Dewey, 1963; Cullen *et al.*, 1980).

Our data contrast with the report by Yuhas *et al.*, (1977), who observed no reduction of radiosensitization in the line 1 carcinoma when WR-2721 was used in combination with MISO. They observed a marked sensitization with 200 mg/kg of MISO (SER = 2.5) even at low X-ray doses: This was not reduced by the addition of 400 mg/kg of WR-2721. However, this carcinoma appears to be an extremely fast growing and radioresistant tumour, since 20 Gy caused growth delay of only one day. If the tumour radioresistance is the result of a large hypoxic fraction, it would explain the sensitization by MISO at low doses, the lack of radioprotection, and the negligible effect of WR-2721 on MISO radiosensitization.

The interaction reported here for tumour radiomodification resembles that which we have previously shown for normal mouse skin (Rojas *et al.*, 1982*b*). In that tissue the WR-2721 radioprotection was significantly reduced in the presence of MISO. A similar effect was observed for oral mucosa (Grigsby & Maruyama, 1981) and for bone marrow (Yuhas *et al.*, 1977), but only a small interaction was seen in skin (Yuhas *et al.*, 1977) and none was reported for salivary glands (Sodicoff *et al.*, 1979). The latter study however, depends upon historical controls which may compromise the conclusions.

We believe that the interaction we have observed is a radiochemical effect rather than a physiological or pharmacokinetic effect because it has also been observed in normal tissues *in vivo* and for cells *in vitro*. The hypothermia that is induced by both drugs in mice could result in changes in blood flow and hence in the oxygenation status of the tumour cells. However the bulk of the experimental studies on hypothermia indicate that the time course for sulphydryl radioprotection does not match the time course for development of hypothermia, and the degree of hypothermia induced by the drug combination would not be sufficient to produce radioprotection through this mechanism. This topic, which was once the subject of considerable controversy, is well summarised by Scott (1963) and Bacq (1965).

The competing effects of oxygen-mimetic compounds and SH compounds have also been widely documented *in vitro* (Chapman *et al.*, 1973; Asquith *et al.*, 1974; Hall *et al.*, 1977, Koch & Howell, 1980, 1981). These data have been interpreted as competition between oxidising and reducing species for fixation and repair of radiation induced radicals, as postulated in the oxygen-fixation hypothesis (Alexander & Charlesby, 1954).

The present data, together with most of the published studies indicate that MISO and WR-2721 are *not* independent in their radiomodifying action, either on tumours or on normal tissues. In addition, there is obviously an interaction in terms of lethal toxicity. Thus, there does not appear to be a firm basis for expecting that the combination of small doses of both drugs will give any therapeutic advantage over their use as single agents. Furthermore, the oxygen dependency of both the radioprotective and radiosensitizing agents means that for clinical relevance, experiments must be performed at appropriate X-ray doses because the population under treatment contains a mixture of euoxic and hypoxic cells. Since the pattern of reoxygenation will determine the proportion of hypoxic cells at the time of each irradiation, more fractionated experiments are needed. Studies performed only with large single doses of X-rays may be misleading.

We are grateful to Professor J.F. Fowler for his help and constructive criticism, to the Cancer Research Campaign for financial support, to Mr. P. Russell and his staff for care of the animals and to Mrs. E. Marriott and Mrs. S. Bloomfield for typing the manuscript.

### References

ADAMS, G.E. (1978). Hypoxic cell sensitizers for radiotherapy. *Int. J. Radiat. Oncol. Biol. Phys.*, **4**, 135.

ALEXANDER, P. & CHARLESBY, A. (1954). In: *Radiobiology Symposium*. (Eds. Bacq & Alexander). London: Butterworths. p. 49.

ALPER, T. (1962). The dependence of chemical protective action on oxygen, as studied with bacteria. *Br. J. Radiol.*, **35**, 361.

ALPER, T. (1979). In: *Cellular Radiobiology*. Cambridge: University Press. p. 87.

ASQUITH, J.C., FOSTER, J.L. & WILLSON, R.L. (1974). Metronidazole ("Flagyl"): A radiosensitizer of hypoxic cells. *Br. J. Radiol.*, **47**, 474.

BACQ, Z.M. (1965). Mechanisms of action: Short lived hypothesis and undeveloped ideas. In: *Chemical Protection against Ionizing Radiation*. (Ed. Kugelmass) Thomas, C.C. p. 180.

BRIDGES, B.A. (1962). Protection of *pseudomonas* sp. against gamma-radiation by dimethyl sulphoxide. *Int. J. Radiat. Biol.*, **5**, 101.

CALDWELL, R.W. & HEIFFER, M.H. (1975). Acute cardiovascular and autonomic effects of WR-2721: A radioprotective compound. *Radiat. Res.*, **62**, 62.

CHAPMAN, J.D., REUVERS, A.P., BORSA, J. & GREENSTOCK, C.L. (1973). Chemical radioprotection and radiosensitization of mammalian cells growing *in vitro*. *Radiat. Res.*, **56**, 291.

CLEMENT, J.J. & JOHNSON, R.K. (1982). Influence of WR-2721 on the efficacy of radiotherapy and chemotherapy of murine tumors. *Int. J. Radiat. Oncol. Biol. Phys.*, **8**, 539.

CONROY, P.J., VON BURG, R., PASSALACQUA, W., PENNY, D.P. & SUTHERLAND, R.M. (1979). Misonidazole neurotoxicity in the mouse: Evaluation of functional pharmacokinetic, electrophysiologic and morphologic parameters. *Int. J. Radiat. Oncol. Biol. Phys.*, **5**, 983.

CULLEN, B.H. MICHALOWSKI, A. & WALKER, H.C. (1980). Correlation between the radiobiological oxygen constant K, and the non-protein sulphydryl content of mammalian cells. *Int. J. Radiat. Biol.*, **38**, 525.

DENEKAMP, J., HIRST, D.G., STEWART, F.A. & TERRY, N.H.A. (1980). Is tumour radiosensitization by misonidazole a general phenomenon? *Br. J. Cancer*, **41**, 1.

DENEKAMP, J., MICHAEL, B.D., ROJAS, A. & STEWART, F.A. (1981). Thiol radioprotection *in vivo*: The critical role of tissue oxygen concentration. *Br. J. Radiol.*, **54**, 1112.

DENEKAMP, J. MICHAEL, B.D., ROJAS, A. & STEWART, F.A. (1982*a*). Radioprotection of mouse skin by WR-2721: The critical influence of oxygen tension. *Int. J. Radiat. Oncol. Biol. Phys.*, **8**, 531.

DENEKAMP, J., ROJAS, A. & STEWART, F.A. (1982*b*). Is radioprotection by WR-2721 restricted to normal tissues? In: *Proceedings of the First Conference on Radioprotectors and Anticarcinogens. Maryland, U.S.A.* (Ed. Nygaard Simic) New York: Plenum Press (in press).

DEWEY, D.L. (1963). The X-ray sensitivity of serratia marcescens. *Radiat. Res.*, **19**, 64.

DISCHE, S., SAUNDERS, M.I., ANDERSON, P. & 6 OTHERS (1978). The neurotoxicity of misonidazole. The pooling of data from 5 centers. *Br. J. Radiol.*, **51**, 1023.

FOWLER, J.F. & DENEKAMP, J. (1979). A review of hypoxic cell radiosensitization in experimental tumors. *Pharmacol. Ther.*, **7**, 413.

GOMER, C.J. & JOHNSON, R.J. (1979). Relationship between misonidazole toxicity and core temperature in C3H mice. *Radiat. Res.*, **78**, 329.

GRIGSBY, P. & MARUYAMA, Y. (1981). Modification of the oral radiation death syndrome with combined WR-2721 and misonidazole. *Br. J. Radiol.*, **54**, 969.

HALL, E.J., ASTOR, M., GEARD, C. & BIAGLOW, J. (1977). Cytotoxicity of R0-0582; enhancement by hyperthermia and protection by cysteamine. *Br. J. Cancer*, **35**, 809.

HARRIS, J.W. & PHILLIPS, T.L. (1971). Radiobiological and Biochemical studies of thiophosphate radioprotective compounds related to cysteine. *Radiat. Res.*, **46**, 362.

HENDRY, J.H. & SUTTON, M.L. (1978). Care with radiosensitizers. *Br. J. Radiol.*, **51**, 927.

HOWES, A.E. (1969). An estimation of changes in the proportions and absolute numbers of hypoxic cells after irradiation of transplanted C3H mouse mammary tumours. *Br. J. Radiol.*, **42**, 441.

KLIGERMAN, M.M., BLUMBERG, A.L., GLICK, J.H., NELSON, D.F., GLOVER, D., YUHAS, J.M., AMOLS, H.I. & GOODMAN, R.L. (1981). Phase 1 trials of WR-2721 in combination with radiation therapy and with the alkylating agents cyclophosphamide and cis-platinum. *Cancer Clin. Trials*, **4**, 469.

KOCH, C.J. & HOWELL, R.L. (1980). Combined radiation-protective and radiation-sensitizing agents. Cysteamine protects against cytotoxicity, radiosensitization and metabolism-enhanced radiosensitization of misonidazole against hypoxic cells. In: *Radiation Sensitizer: Their Use in the Clinical Management of Cancer*. (Ed. Brady). Masson Publ., p. 239.

KOCH, C.J. & HOWELL, R.L. (1981). Combined radiation-protective and radiation-sensitizing agents. II. Radiosensitivity of hypoxic or aerobic Chinese hamster fibroblasts in the presence of cysteamine and misonidazole: Implications for the "oxygen effect" (with appendix on calculations of dose modifying factors). *Radiat. Res.*, **87**, 265.

PHILLIPS, T.L., KANE, L. & UTLEY, J.F. (1973). Radioprotection of tumor and normal tissues by thiophosphate compounds. *Cancer*, **32**, 528.

ROJAS, A., STEWART, F.A. & DENEKAMP, J. (1982*a*). Experimental radiotherapy with WR-2721 and misonidazole. *Int. J. Radiat. Oncol. Biol. Phys.*, **8**, 527.

ROJAS, A., STEWART, F.A. & DENEKAMP, J. (1982*b*). Interaction of radiosensitizers and WR-2721. 1. Modification of skin radioprotection. *Br. J. Cancer*, **45**, 684.

SCOTT, O.C.A. (1963). The modification of tissue response to radiation injury. *Ann. Rev. Med.*, **14**, 371.

SHELDON, P.W. & HILL, S.A. (1977). Hypoxic cell radiosensitizers and local control by X-ray of a transplanted tumour in mice. *Br. J. Cancer*, **35**, 795.

SODICOFF, M., CONGER, A.D., PRATT, N.E., SINESI, M. & TREPPER, P. (1979). Chemoprotection of the rat parotid gland by combined use of WR-2721 and R0-07-0582. *Radiat. Res.*, **80,** 348.

STEWART, F.A., DENEKAMP, J. & RANDHAWA, V.S. (1982*a*). Skin sensitization by misonidazole: A demonstration of uniform mild hypoxia. *Br. J. Cancer,* **45,** 869.

STEWART, F.A., ROJAS, A. & DENEKAMP, J. (1982*b*). Radioprotection of two mouse tumours by WR-2721 in single and fractionated treatments. *Int. J. Radiat. Oncol. Biol. Phys.* (in press).

UTLEY, J.F., PHILLIPS, T.L., KANE, L.J., WHARAM, M.D. & WARA, W.M. (1974). Differential radioprotection of euoxic and hypoxic mouse mammary tumors by a thiophosphate compound. *Radiology,* **110,** 213.

WRIGHT, E.A. (1962). The influence of combining hypoxia and cysteamine treatments on whole body irradiation of mice. *Br. J. Radiol.*, **35,** 361.

YUHAS, J.M., PROCTOR, J.O. & SMITH, L.H. (1973). Some pharmacologic effects of WR-2721: Their role in toxicity and radioprotection. *Radiat. Res.*, **54,** 222.

YUHAS, J.M., YURCONIC, M., KLIGERMAN, M.M., WEST, G. & PETERSON, D.F. (1977). Combined use of radioprotective and radiosensitizing drugs in experimental radiotherapy. *Radiat. Res.*, **70,** 433.

YUHAS, J.M. (1980). Active versus passive absorption kinetics as the basis for selective protection of normal tissues by S-2-(3-aminopropylamino) ethylphosphorothioic acid. *Cancer Res.*, **40,** 1519.

YUHAS, J.M. (1981). On the potential application of radioprotective drugs in solid tumor radiotherapy. In: Radiation–Drug Interactions in the Treatment of Cancer. (Eds. Sokol & Maickel) New York: John Wiley & Sons. p. 113.